# Achievements in the Pathophysiology and Treatment of Insulin Resistance: Every Step Matters

**DOI:** 10.3390/nu17071223

**Published:** 2025-03-31

**Authors:** George Dimitriadis

**Affiliations:** 2nd Department of Internal Medicine, Research Institute and Diabetes Center, National and Kapodistrian University of Athens Medical School, 15772 Athens, Greece; gdimitr@med.uoa.gr; Tel.: +30-6944250718

Insulin regulates glucose, lipid, and protein metabolism in insulin-sensitive tissues. In the liver, insulin stimulates glycogen synthesis while inhibiting glycogenolysis and gluconeogenesis, which reduces endogenous glucose production. In muscle, insulin promotes glucose uptake and metabolism while inhibiting glycogen breakdown. In adipose tissue, insulin increases lipid synthesis and suppresses lipolysis, leading to a decreased release of non-esterified fatty acids (NEFA)—potent inhibitors of glucose uptake in muscle and stimulators of liver gluconeogenesis—and glycerol, an important substrate for gluconeogenesis. Additionally, insulin promotes protein synthesis and limits proteolysis, lowering plasma amino acid levels, which are important substrates for gluconeogenesis. Lastly, insulin stimulates nitric oxide production in the vascular endothelium of muscle and adipose tissue, enhancing blood flow in both the micro- and macro-vasculature. These effects work together to maintain metabolic homeostasis under various health and disease conditions [1].

In the postabsorptive (fasting) state, the liver is the primary site for glucose production and the maintenance of euglycemia, while skeletal muscle plays a minor role. In the postprandial state, muscle is primarily responsible for removing glucose from peripheral circulation, which is aided by suppressing lipolysis and NEFA production from adipose tissue (Figure 1) [1].

Type 2 diabetes (T2D) is a metabolic disease triggered by obesity and a sedentary life in people with a genetic predisposition. The resistance of insulin-responsive tissues to insulin (liver, muscle, adipose tissue, and vascular endothelium) is a primary pathophysiological mechanism accompanied by β-cell insufficiency and metabolic dysregulation (2). The prevention of T2D and the efforts for therapeutic interventions following diagnosis have dominated the world literature for a long time in attempts to avoid chronic microvascular and macrovascular complications that shorten lifespan. Since the discovery of insulin in 1922 and with the contribution of molecular biology over the last few decades, there has been great progress in elucidating insulin action, how it affects signal transduction in insulin-sensitive tissues, and the mechanisms behind the development of insulin resistance, leading to the evolution of new therapeutic modalities for T2D [2,3]. Innovative treatments such as daily and weekly GLP-1 receptor agonists (GLP-1RAs) [4], SGLT-2 inhibitors (SGLT-2is) [5], and weekly insulins [6] have dramatically changed the therapeutic landscape by improving clinical care and outcomes and decreasing the risk and severity of cardiovascular and chronic kidney disease.

Based on this background, the Special Issue “*Insulin 100th Anniversary: Century of Innovation for Diabetes*” includes eleven publications (four reviews and seven original articles) covering several topics on metabolism, pathophysiology, and treatment of T2D such as protein/ketone body metabolism, β-cell dysfunction in early/youth- and adult-onset T2D, time-restricted feeding and insulin resistance, dietary issues and diabetic nephropathy, lifestyle patterns, prediabetes, risk of overt T2D, gestational diabetes, blood pressure patterns, insulin resistance, diabetic peripheral neuropathy, GLP-1RAs and depression, and SGLT2is and the inflammasome.

In a comprehensive review article, Paolo Tessari [7] provided a synthetic and historical view of the key steps taken from the discovery of insulin as an “anabolic hormone” to the analysis of its effects on amino acid metabolism and protein synthesis, as well as of its interaction with nutrients at the whole-body, organ/tissue, and molecular level.

In another review article, Veneti, S. et al. [8] analyzed the role of ketones and ketogenic diets in diabetes. The authors provided new evidence suggesting that ketone bodies are involved in the diagnosis, treatment, and complications of diabetes. Although ketone diets appear to be promising new additions to medical nutrition therapy regimes for the treatment of obesity and T2D, further research is required to support their adoption.

In an original article, Malodobra-Mazur, M. et al. [9] investigated the hypothesis that natural compounds from plant extracts could increase insulin sensitivity in the adipose tissue. The authors analyzed the effects of phospholipid derivatives of selected natural aromatic acids on insulin action in 3T3-L1 adipocytes rendered insulin resistant via incubation with palmitic acid. The results showed that cinnamic acid and 3-methoxycinnamic acid restored the sensitivity of glucose uptake to insulin in the adipocytes, suggesting that these compounds may have potential use in the prevention or treatment of T2D.

In a narrative review, Serbis et al. [10] summarized all the available evidence on the mechanisms involved in defective insulin secretion in early/youth-onset and adult-onset T2D and the role of nutrients in this process. As the authors point out, relative information on early-onset T2D is scarce compared to adult-onset T2D. A chronic imbalance between caloric intake and expenditure, along with deficient micronutrient and vitamin intake—such as zinc and vitamin C—can lead to weight gain, insulin resistance, and β-cell failure/reduced insulin secretion, accelerating the development of clinical T2D in genetically susceptible individuals. In younger people with T2D, severe β-cell dysfunction—even at the time of diagnosis—along with its rapid decline, makes early-onset T2D an aggressive type of this disease.

In addition to the sleep/wake cycle and physical activity, chrononutrition significantly influences the coordination between the central and peripheral biological clocks and the circadian rhythms of hormones and neurotransmitters, contributing to optimal metabolic regulation. A lack of synchronization within the circadian system can lead to metabolic dysregulation, increasing the risk of metabolic diseases such as obesity and T2D [11]. Innovative nutritional strategies, such as time-restricted feeding (TRF), aligned with the circadian system, have been proposed for weight loss, the regulation of postprandial hyperglycemia, and enhanced insulin sensitivity. In a systematic review, Tsitsou et al. [12] examined the effects of TRF compared to existing nutritional treatments on body weight loss and composition, postprandial glucose responses, and insulin resistance in otherwise healthy overweight or obese individuals, as well as those with prediabetes or T2D. A clinically significant weight loss of more than 5% was achieved through TRF combined with caloric restriction. Furthermore, TRF can improve postprandial glycemic responses/glucose variability and insulin sensitivity.

In an original article, Mourouti et al. [13] investigated the association of different lifestyle patterns (LPs) with the development of prediabetes in 2759 adults genetically susceptible to T2D in six European countries (Feel4Diabetes study). Two LPs were used: LP1 was characterized by high physical activity, the consumption of breakfast, high consumption of fruits, berries, vegetables, nuts, and seeds, and low consumption of salty snacks/soft drinks with sugar. LP2 was characterized by low physical activity and high consumption of salty and sweet snacks, soft drinks with sugar, and juice with sugar. After adjusting for confounders, in contrast to LP1, LP2 was highly associated with the development of prediabetes.

Depression is a common and serious comorbidity linked to Metabolic Syndrome, T2D, and insulin resistance [14]. GLP-1RAs, a potent class of medications for treating T2D and obesity, promote weight loss, enhance insulin sensitivity through direct and indirect mechanisms, and provide neuroprotective effects in the central nervous system [4]. In this Special Issue, Yang et al. [15] conducted a study to explore whether the GLP-1RA exendin-4 could reduce depression-like behaviors in db/db mice with diabetes. The activation of GLP-1 receptors by exendin-4 decreased microglial inflammation by improving mitochondrial function and reducing the production of reactive oxygen species and oxidative stress. The authors concluded that GLP-1RAs may represent a promising therapy for depression related to T2D.

Inflammasomes, particularly NLRP3, appear to play a significant role in mediating inflammation in individuals with T2D, especially those with chronic complications such as heart failure, chronic kidney disease, and cognitive impairment, increasing their progression rates [16]. SGLT-2is enhance urinary glucose excretion and natriuresis, improve hyperglycemia, reduce glucotoxicity and oxidative stress, promote weight loss, alleviate inflammation, and increase insulin sensitivity through indirect mechanisms; these agents are widely utilized in the treatment of T2D [5]. In a review article, Kounatidis et al. [17] presented evidence showing that SGLT-2is can reduce the activation of the NLRP3 inflammasome in the kidney, heart, and neurons, thereby diminishing inflammation and progression rates.

Diabetic peripheral neuropathy (DPN) is a chronic microvascular complication of diabetes primarily linked to hyperglycemia, but it is also associated with insulin resistance [18]. The role of hypertension, which is also related to insulin resistance, in the pathogenesis of DPN remains unclear. In an original article, Ntavidi et al. [19] examined the relationship between DPN and hypertension in adults with diabetes. The findings did not support a correlation between hypertension and DPN. However, in contrast to participants with a normal dipping status (a typical 10–20% decrease in blood pressure during sleep compared to daytime measurements), non-dippers (those who do not exhibit the usual nighttime drop in blood pressure, which remains elevated throughout the 24 h) faced a four-fold higher risk of developing medium-to-severe DPN.

Diabetic nephropathy (DN) is the leading cause of chronic kidney disease. Optimizing nutrition may be a significant and modifiable factor that could prevent or delay the onset of DN [20]. In a cross-sectional analysis, Lin et al. [21] recruited patients with DN (urinary albumin–creatinine ratio > 30 mg/g) to investigate their dietary intake compared to participants with diabetes who did not have DN. The results indicated that a diet rich in high-fat fish, shellfish, and soybean products, along with a lower Σn-6/Σn-3 PUFAs (polyunsaturated fatty acids) ratio, can reduce the risk of DN.

Gestational diabetes (GD) develops after the first trimester of pregnancy, with insulin resistance in insulin-responsive tissues being the primary pathophysiological mechanism. Weight gain during pregnancy signifies maternal fat accumulation, a vital aspect of fetal growth and healthy pregnancy progression [22]. Tranidou et al. [23] recruited 5948 pregnant women, of whom 5.5% developed GD. The risk of developing GD was positively associated with weight gain during pregnancy. This association was stronger in individuals who were already obese at the beginning of pregnancy and in those who became pregnant at an older age.

This Special Issue has gathered information on several topics related to insulin resistance and the treatment and complications of T2D. Due to the rapid advancements in understanding insulin action, challenges in treating T2D and preventing chronic complications are expected to be resolved.

## Figures and Tables

**Figure 1:**
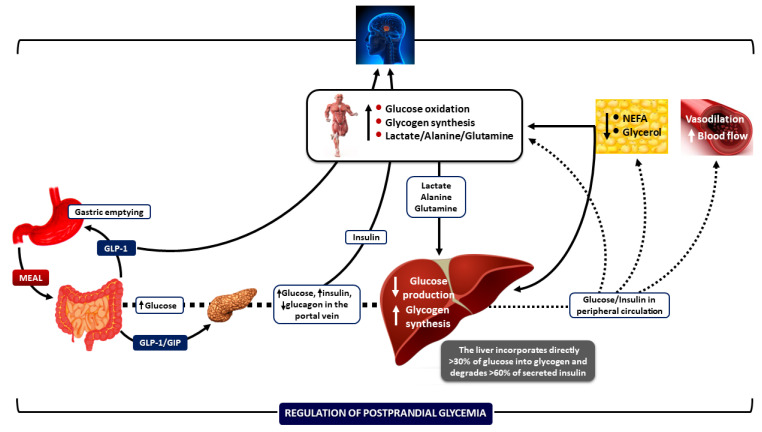
Integrative mechanisms for regulating postprandial hyperglycemia during meal ingestion. Several processes work together—such as gastric emptying, intestinal glucose absorption, the secretion and action of gastrointestinal hormones, hyperglycemia mass action effects, and the secretion and action of insulin and glucagon—to ensure optimal regulation of blood glucose fluctuations after meals. Increased insulin concentrations promote lipid synthesis in the adipose tissue and inhibit lipolysis, lowering blood NEFA levels. This change helps reduce endogenous glucose production, enabling glucose storage as glycogen and enhancing glucose uptake by skeletal muscle. Insulin also increases blood flow rates in muscle and adipose tissue to support glucose uptake and facilitate the removal of lipids from the peripheral circulation, respectively. CNS: central nervous system; NEFA: non-esterified fatty acids; GIP: glucose-dependent insulinotropic polypeptide; and GLP-1: glucagon-like peptide-1. Downward arrows depict a decrease; upward arrows depict an increase. (Reproduced with modifications from reference [1] [Dimitriadis, GD et al., Nutrients **2021**, 13, 159, https://doi.org/10.3390/nu13010159]).
